# Pitfalls of immunotherapy: lessons from a patient with CTLA-4 haploinsufficiency

**DOI:** 10.1186/s13223-018-0272-7

**Published:** 2018-10-22

**Authors:** Leisa Rebecca Watson, Charlotte A. Slade, Samar Ojaimi, Sara Barnes, Pasquale Fedele, Prudence Smith, Justine Marum, Sebastian Lunke, Zornitza Stark, Matthew F. Hunter, Vanessa L. Bryant, Michael Sze Yuan Low

**Affiliations:** 1 Faculty of Medicine, Nursing and Health Sciences, Monash University, Clayton, VIC Australia; 2 Immunology Division, Walter and Eliza Hall Institute of Medical Research, 1G Royal Parade, Parkville, VIC Australia; 3 Department of Medical Biology, The University of Melbourne, Parkville, VIC Australia; 4 Department of Clinical Immunology and Allergy, The Royal Melbourne Hospital, Parkville, VIC Australia; 5 Department of Immunology and Allergy, Monash Health, 246 Clayton Road, Clayton, VIC Australia; 6 Monash Haematology, Monash Health, 246 Clayton Road, Clayton, VIC Australia; 7 Victorian Clinical Genetics Service, Murdoch Children’s Research Institute, 50 Flemington Road, Parkville, VIC Australia; 8 Department of Pathology, The University of Melbourne, Parkville, VIC Australia; 9 Department of Paediatrics, The University of Melbourne, Parkville, VIC Australia; 10 Monash Genetics, Monash Health, 246 Clayton Road, Clayton, VIC Australia; 11 Department of Paediatrics, Monash University, Clayton, VIC Australia

**Keywords:** Immunotherapy, Daclizumab, CD25, IL-2, T regulatory cell, Multiple sclerosis, Autoimmune, Primary immunodeficiency, CTLA-4 haploinsufficiency with autoimmune infiltration, Cytotoxic lymphocyte antigen 4

## Abstract

**Background:**

Daclizumab is a humanized monoclonal antibody that blocks CD25, the high affinity alpha subunit of the interleukin-2 receptor. Daclizumab therapy targets T regulatory cell and activated effector T cell proliferation to suppress autoimmune disease activity, in inflammatory conditions like relapsing and remitting multiple sclerosis. Here, we present the first report of agranulocytosis with daclizumab therapy in a patient with relapsing and remitting multiple sclerosis.

**Case presentation:**

Our patient was a 24-year-old Australian female with a clinical history of atopy, lymphocytic enteritis complicated by B12 deficiency, relapsing and remitting multiple sclerosis, recurrent lower respiratory tract infections, vulval/cervical intraepithelial neoplasia and melanoma. She was commenced on daclizumab therapy after failing several lines of treatment for relapsing and remitting multiple sclerosis. During a hospital admission for lymphocytic enteritis, she was incidentally diagnosed with combined immunodeficiency with hypogammaglobulinaemia and declined proposed regular intravenous immunoglobulin infusions. Following six months of daclizumab therapy, our patient presented to hospital with febrile neutropenia. No clear infective cause was found, despite numerous investigations. However, bone marrow biopsy revealed agranulocytosis with an apparent maturation block at the myeloblasts stage. Neustrophil recovery occurred following cessation of daclizumab and the initiation of T cell immunosuppressive agents including systemic corticosteroids and methotrexate. The patient was further investigated for combined immunodeficiency and whole exome sequencing revealed a novel heterozygous missense variant in *cytotoxic T lymphocyte antigen 4* (*CTLA4*), leading to a diagnosis of CTLA-4 haploinsufficiency with autoimmune infiltration (CHAI).

**Conclusion:**

This case demonstrates that autoimmune disease may be the presenting feature of primary immunodeficiency and should be appropriately investigated prior to the commencement of immunotherapy. Genetic clarification of underlying primary immunodeficiency may provide critical clinical information that alters the safety of the proposed treatment strategy.

## Background

Daclizumab is a humanized monoclonal antibody that blocks CD25, the high affinity alpha subunit of the interleukin-2 receptor (IL-2R). CD25 is constitutively expressed by T regulatory cells and upregulated by activated T and B lymphocytes to enhance autocrine interleukin-2 (IL-2) signaling. The cytokine IL-2 stimulates the clonal expansion of activated effector lymphocytes and is essential for T regulatory cell proliferation and survival [1]. Daclizumab was approved in 2016 for the treatment of relapsing and remitting multiple sclerosis (RRMS), an autoimmune demyelinating disease of the central nervous system in which T cells play a central role. Daclizumab inhibits mature dendritic cell presentation of CD25 to antigen-specific T cells, thereby inhibiting T cell proliferation and reducing nervous inflammation in multiple sclerosis [2]. Here we present a patient with RRMS who developed agranulocytosis following daclizumab therapy and who was subsequently found to have a novel mutation in the gene encoding the inhibitory immune receptor cytotoxic T lymphocyte antigen-4 (CTLA-4).

## Case presentation

**Fig. 1:**
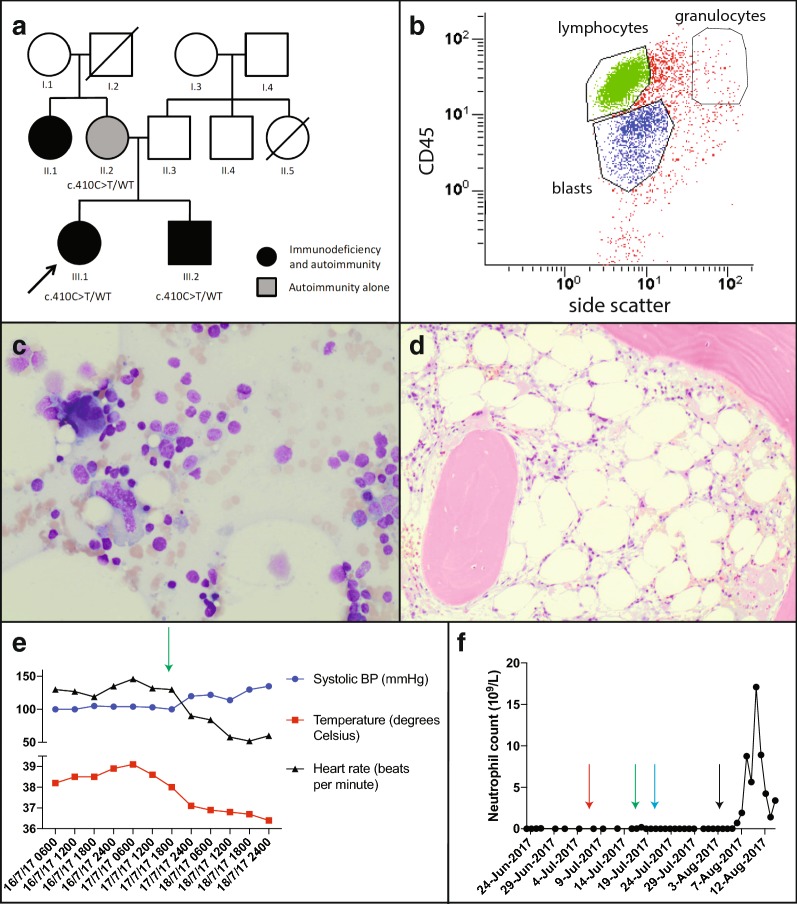
**a** Family pedigree: arrow indicates proband. Circles represent females, squares represent males; strikethrough represent deceased individuals, clinical conditions as outlined in key. **b** Flow cytometry of bone marrow aspirate demonstrating agranulocytosis with almost no cells in the granulocytic gate; lymphocytes are gated in green and blasts gated in blue. Bone marrow aspirate (**c**) and trephine (**d**) demonstrating a hypocellular marrow with adequate erythroid and megakaryocytic precursors but no maturing granulopoiesis. **e** Rapid resolution of tachycardia and fevers on commencement of systemic corticosteroids (green arrow). **f** Longitudinal neutrophil counts during course of admission with commencement of G-CSF (red arrow), corticosteroids (green arrow), oral methotrexate (blue arrow) and subcutaneous methotrexate (black arrow)

Our patient was a 24-year-old Australian female with a clinical history of atopy with childhood asthma, eczema and allergic rhinitis, and lymphocytic enteritis complicated by B12 deficiency diagnosed age 23. Genetic studies revealed the patient is HLA DQ2/8 negative and her enteritis was successfully treated with oral budesonide. She also manifested immunodeficiency with recurrent lower respiratory tract infections (age 23), vulval/cervical intraepithelial neoplasia (age 22) and melanoma (age 24), some of which preceded her immunosuppressed state. The family history was notable for combined variable immunodeficiency (CVID) in her brother and maternal aunt, both with autoimmune features, and her mother had alopecia (Fig. 1a; Tables 1, 2). Despite the above characteristics, the patient and her family had no formal diagnosis but had been referred for review by a specialist immunologist.

**Table 1:** Clinical features of kindred

Individual	III.1 (proband)	III.2	II.1	II.2	I.1
Birth year	1993	1995	N/A	1975	N/A
Diagnosis (age)	CHAI (23)	CHAI (22)	N/A	CHAI (43)	N/A
Autoimmune/infective manifestations (age at onset, if known)	RRMS (19) ILD (21) HGG (23) Melanoma (23) Recurrent LRTI (23) HPV: CIN II/VIN III Villous atrophy Lymphocytic colitis (22) Autoimmune cytopenia ITP (21), neutropenia (23)	Lymphocytic panenteritis (11) Autoimmune thyroiditis (~ 17) HGG (17) SPI HPV: Warts Herpes zoster	HGG SPI Alopecia universalis (16) ILD NHL	Chronic sinusitis (turbinectomy 45) Transient GIT disturbances—no diagnosis (40) Alopecia universalis (29)	Presumed spongiform encephalopathy (77)
Other manifestations	B12 deficiency (23) Osteoporosis (24) Allergic rhinitis (childhood) Asthma (childhood) Eczema (childhood)	Osteopenia Delayed puberty Eczema Acne	Presumed drug-induced cardiomyopathy Asthma	Asthma Allergic rhinitis	NA
Histological features	Enteric biopsies: intraepithelial lymphocytosis and villous atrophy	Gastric/colonic: panenteric lymphocytic infiltration with flattening of villus architecture.	N/A	N/A	N/A
Treatments	Methotrexate—current Prednisolone—current IVIg—current Fingolimod (ITP) Natalizumab (ILD) Daclizumab (neutropenia)	6-Mercaptopurine: pancreatitis IVIg—current	N/A	Nil	N/A

*BM* bone marrow, *CID* combined immunodeficiency, *CHAI* CTLA-4 haploinsufficiency with autoimmune infiltration, *CIN II* cervical intraepithelial neoplasia grade II, *GIT* gastrointestinal tract, *HGG* hypogammaglobulinaemia, *HPV* human papilloma virus, *ILD* interstitial lung disease, *ITP* immune thrombocytopenia, *IVIg* intravenous immunoglobulin, *LRTI* lower respiratory tract infections, *MS* multiple sclerosis, *N/A* not available, *NHL* non-hodgkin lymphoma, *SPI* sinopulmonary infections, *T-regs* regulatory T-cells (CD4^+^CD25^+^CD127neg), *sCD25* soluble CD25/IL22R alpha, *VIN III* vulval intraepithelial neoplasia grade III

**Table 2:** Immunological testing

Test	III.1 (proband)	III.2	II.2
IgG (7.5–15.6 g/L)	4.9	5.7	10.1
IgA (0.8–4.5 g/L)	< 0.1	0.5	3.0
IgM (0.4–3.0 g/L)	0.8	0.1	0.5
IgE (0–160 KU/L)	< 1		
CD4 T-cell			
(0.4–1.6 × 10^9^/L)	0.2 × 10^9^/L	0.4 × 10^9^/L	0.4 × 10^9^/L
(31–59%)	57%	51%	52%
CD8 T-cell			
(0.2–0.9 × 10^9^/L)	0.1 × 10^9^/L	0.2 × 10^9^/L	0.2 × 10^9^/L
(12–42%)	29%	27%	21%
B-cells			
(0.1–0.6 × 10^9^/L)	0.03 × 10^9^/L	0.07 × 10^9^/L	0.1 × 10^9^/L
(6–26%)	7%	9%	13%
NK-cells			
(0.06–0.8 × 10^9^/L)	0.02 × 10^9^/L	0.04	0.08 × 10^9^/L
(7–28%)	5%	5%	11%
Switched memory B-cells			
No RR	0.0 × 10^9^/L	0.00 × 10^9^/L	2 (5–62 cells/μL)^a^
(6.5–29.2%)	0.4%	1.9%	0.33% (0.4–3.3%)^a^
CD21^lo^ cells			
No RR	27%	17%	N/A
Naïve T-cells			
(0.1–0.6 × 10^9^/L)	0.06 × 10^9^/L	0.21 × 10^9^/L	N/A
(26–60%)	21%	58.5%	
T-cell proliferation			
(patient vs control)	27% vs 59.6%	N/A	N/A
Regulatory T-cells			
(0.025–0.180 × 10^9^/L)	0.009 × 10^9^/L	0.014 × 10^9^/L	0.019 × 10^9^/L
(4–17%)	3.2%	4.3%	5.1%
Soluble CD25			
(186–2678 pg/mL)	5307	5753	N/A

^a^Tested in a different laboratory, reference ranges noted

Five years prior to her current presentation our patient was diagnosed with RRMS at age 19 in the setting of recurrent optic neuritis and demyelinating lesions on MRI (Table 1). She had progressed through several lines of RRMS therapy including dimethyl fumarate, fingolimod and natalizumab. Dimethyl fumarate was discontinued due to lymphopaenia (age 20) and fingolimod was ceased due to immune thrombocytopenic purpura (ITP) (age 22), a reported complication of fingolimod therapy [3]. Natalizumab therapy was also discontinued, due to presumed natalizumab-induced interstitial lung disease (age 22) [4]. This diagnosis was supported by bilateral patchy nodular infiltration with ground glass opacities and interlobular septal thickening on high resolution computed tomography, bronchoscopy and biopsy that showed no granulomatous inflammation or features of malignancy. The patient was commenced on daclizumab therapy.

Following 3 months of daclizumab therapy, our patient presented to hospital with an exacerbation of lymphocytic enteritis and was given a diagnosis of combined immunodeficiency with hypogammaglobulinaemia, reduced B cells and switched memory B cells with absent pneumococcal vaccine responses; T cell lymphopenia with reduced naïve T-cells (Table 2). The patient initially declined regular intravenous immunoglobulin (IVIg) infusions to treat hypogammaglobulinaemia.

Six months following commencement of daclizumab therapy, our patient presented to hospital with a 1-week history of nausea, vomiting, tachycardia and high fevers (> 39 °C). Further assessment revealed an absolute neutropaenia (0.0 × 10^9^/L) and elevated C-reactive protein. No clear infective cause was found despite numerous investigations, including blood, bone marrow, urine and stool cultures. Bone marrow biopsy revealed an agranulocytosis with an apparent maturation block at the myeloblasts stage but no features of hemophagocytosis and Epstein–Barr virus (EBV) in situ hybridization was negative (Fig. 1b–d).

Daclizumab was suspected to be the underlying cause of agranulocytosis and was ceased. However, there was minimal improvement for several weeks despite cessation of daclizumab and treatment with broad spectrum antibiotics and antivirals, IVIg and the neutrophil granulopoiesis stimulant, granulocyte-colony stimulating factor (G-CSF). On commencement of T cell immunosuppressive agents including systemic corticosteroids and methotrexate, the patient made a rapid recovery with resolution of fever and agranulocytosis within 3 weeks (Fig. 1e, f). The patient has not received any further daclizumab therapy and remains well on low dose prednisolone and methotrexate.

Whole-exome sequencing was performed in the proband and her brother. A novel heterozygous missense variant in *CTLA4* was identified (c.410C > T; p.Pro137Leu in exon 2 of *CTLA4*; Fig. 1a). Their mother was also confirmed to carry the mutation, and the aunt is awaiting clinical testing. This novel variant affects the CTLA-4 protein at a highly conserved amino acid in an essential functional domain and is classified as ‘likely pathogenic’. No additional mutations were identified in our patient.

## Discussion and conclusions

CTLA-4 is an inhibitory receptor constitutively expressed by T regulatory cells that suppresses effector T cell proliferation [5]. CTLA-4 haploinsufficiency in humans causes T cell hyperproliferation with peripheral lymphopaenia, reduced B cell tolerance, survival and hypogammaglobulinaemia [6]. Individuals with CTLA-4 haploinsufficiency exhibit variable clinical penetrance and phenotype [6, 7]. In this patient, CTLA-4 haploinsufficiency caused a combined phenotype of multiple autoimmune diseases (RRMS, chronic lymphocytic enteritis and interstitial lung disease) in addition to neoplastic events and a susceptibility to autoimmune cytopenias demonstrated in response to immune-modulating drugs. Enteropathy, hypogammaglobulinaemia, respiratory infections and solid cancers are common in patients with CTLA-4 haploinsufficiency, consistent with this patient’s phenotype [7]. Our patient was subsequently given a diagnosis of CTLA-4 haploinsufficiency with autoimmune infiltration (CHAI) (Table 1).

T regulatory cells are essential for the modulation of effector T cell responses to self-antigens and an absence of T regulatory cells predisposes to extensive autoimmune disease [8]. Our patient’s CTLA-4 mutation produced reduced proportions of T regulatory cells, and likely conveyed a defect in T regulatory cell suppressive function, as seen in human studies of CTLA-4 mutations [7]. In this case, targeting IL-2 signaling via administration of daclizumab has caused further insult to an already diminished T regulatory cell response. The identification of the genetic cause of the patient’s condition, through clinical genomic sequencing, provides an opportunity for targeted precision therapy. Abatacept, a humanized CTLA4-IgG fusion protein which has been successfully used in patients with CTLA4 haploinsufficiency [9], and other therapies such as mTOR inhibitors and allogeneic bone marrow transplantation are currently being considered in our patient.

Agranulocytosis in response to daclizumab therapy has not been reported previously. We hypothesize that inadequate T regulatory cell function with the specific blockade of CD25 by daclizumab and our patient’s CTLA-4 haploinsufficiency led to uncontrolled autoimmune destruction of neutrophils by hyper-proliferative effector T cells. We are unable to prove a direct causative effect for daclizumab; with autoimmune neutropenia not excluded; however, given the known dependence on IL-2 for T regulatory cell function [1], this case demonstrates the potential for serious consequences following CD25 blockade in patients with CID and in particular, those with dysfunctional T regulatory cells due to defects altering CTLA-4 function. Furthermore, this case highlights the importance of considering an underlying primary immunodeficiency in the context of autoimmune disease; especially in the context of neoplastic disease and a family history of CVID or autoimmunity; before commencing immunosuppressive therapy. In patients with primary immunodeficiency, genetic diagnosis has great potential to clarify the pathogenesis of underlying primary immunodeficiency, protect the patient from unanticipated harm from immunotherapy and reveal opportunities for personalized therapies and precision medicine in these challenging conditions.

## Abbreviations

CHAI: CTLA-4 haploinsufficiency with autoimmune infiltration
CVID: combined variable immunodeficiency
CID: combined immunodeficiency
CTLA-4: cytotoxic lymphocyte antigen 4
EBV: Epstein–Barr virus
G-CSF: granulocyte-colony stimulating factor
IL-2: interleukin 2
IL-2R: interleukin 2 receptor
IVIg: intravenous immunoglobulin
ITP: immune thrombocytopenic purpura
MRI: magnetic resonance imaging
RRMS: relapsing and remitting multiple sclerosis
